# Migration of Excitation Energy in Furocoumarins

**DOI:** 10.3389/fchem.2021.754950

**Published:** 2021-11-04

**Authors:** O.N. Tchaikovskaya, N.G. Dmitrieva, E.N. Bocharnikova, V.S. Chaidonova, P.V. Avramov

**Affiliations:** ^1^ Laboratory Photophysics and Photochemistry of Molecules, Department of Physics, Tomsk State University, Tomsk, Russia; ^2^ Laboratory of Quantum Electronics, The Institute of Electrophysics of the Ural Division of the Russian Academy of Sciences, Ekaterinburg, Russia; ^3^ Department of Medical Biology, Siberian State Medical University, Tomsk, Russia; ^4^ Laboratory of Physical and Chemical Methods, Hygienic and Epidemiological Center in Republic of Khakassia, Abakan, Russia; ^5^ Department of Chemistry, Kyungpook National University, Daugu, South Korea

**Keywords:** furocoumarin, luminescence, singlet-singlet transition, triplet-triplet transition, photodynamic activity

## Abstract

The migration of excitation energy of a number of psoralen compounds has been studied. For this, the methods of induced absorption spectroscopy, stationary electron spectroscopy, fluorescence and phosphorescence, as well as quantum chemistry were used. A comparative photostability of psoralen was achieved by exposure to a XeCl excilamp irradiation (emission wavelength λ_em_ = 308 nm) with parameters Δλ = 5–10 nm, W_peak_ = 18 mW/cm^2^, *p* = 8.1 J/cm^3^, f = 200 kHz, pulse duration 1 μs. It was found that the singlet-triplet transition played a major role in the migration of excitation energy into triplet states. Among all tested compounds, substances with an OCH_3_-group in the structure have the strongest effect on the spectral-luminescent characteristics.

## Introduction

Due to its sensitivity, fluorescence spectroscopy (Weber et al., 2020; Keuler et al., 2021) has become one of the most commonly used methods in biomedical research. Coumarin-based sensors hold great promise in detecting residual amounts of heavy metals in the body (Wei et al., 2018). Currently, there is an active search for anticancer drugs (Shen et al., 2019; Spreckelmeyer et al., 2018). Due to the unfavorable activity and selectivity of tumor cells, the number of inhibitors is very limited and their effect remains unknown. The authors of the work present studies of an anticancer inhibitor (Bai et al., 2021) based on a coumarin scaffold and low molecular weight phenolic compounds and show its therapeutic effect in the treatment of cancer by disrupting tubulin polymyrization. More and more attention is paid to chemotherapy of cancer cells that respond to the redox potential. The chemotherapeutic molecule attaches to the fluorophore through a self-disrupting linker (Odyniec et al., 2019). There is an active search for a “fluorescent linker” that can be both a diagnosis and a therapeutic agent. Such a theranostic prodrug becomes possible to create on the basis of a self-destructive coumarin linker. The wide possibilities for the synthesis of various coumarin derivatives using virtual combinatorial chemistry and spectrophotometry allowed the authors (Rauhamäki et al., 2018) to create a powerful low molecular weight cancer inhibitor based on 3-phenylcoumarin. The new compounds were found to cause >70% inhibition at a concentration of 100 nM to 1 μM, and 6-methoxy-3-(4-(trifluoromethyl) phenyl)-2H-chromen-2-one at a concentration of approximately 56 nM. At the same time, without any substituents, 3-phenylcoumarin has no biological effect. In (Ibrar et al., 2018), it was shown that in the treatment of Alzheimer’s disease, the effective role of coumarinylthiazoles and oxadiazoles is to inhibit the hydrolysis of acetylcholine in cholinergic synapses, blocking its metabolic activity. Scientists in developed countries are trying to find a solution as soon as possible, working on the creation of vaccines and antiviral drugs. The authors (Yañez et al., 2021) evaluated compounds of coumarin and quinoline derivatives as promising SARS-CoV-2 Mpro inhibitors.

Furocoumarins are heterocyclic aromatic compounds resulting from the condensation of a furan ring with a coumarin ring (Gasparro, 1996), as well as classic chemical compounds with phototoxic properties that naturally occur in many plants (Dolan et al., 2010; Wagstaff, 1991). Furocoumarins have attracted close attention of researchers in recent decades due to their photoactivity. Contact with exposure to ultraviolet radiation can lead to skin burns, a reaction known as phytophotodermatitis (Lagey et al., 1995). The emergence of phytophotodermatitis among therapeutic agents to increase melanin production and improve resistance to sunlight (Scott et al., 1976). Furocoumarins have also been used to treat vitiligo, psoriasis, and other skin conditions with psoralen and ultraviolet (or PUVA) therapy (Parrish et al., 1974; Stern, 2012). Furocoumarin therapy involves topical or oral administration of a furocoumarin derivative, usually 8-methoxypsoralen, followed by irradiation for 1–2 h with near-UV light (about 320–400 nm). These compounds are of particular interest because they have good spectral characteristics capable of accumulating in tissues in high qualities, as well as, in most cases, high photodynamic activity, which allows them to be used as promising photosensitizers for biology and medicine. Using these opportunities for use. Interestingly, due to the ability of furocoumarins to interact and disrupt DNA replication, there is great interest in the development of anti-cancer therapies. Early *in vitro* studies have already shown that furocoumarins can inhibit the growth of various cell types, including cancer and non-small cell lung cancer (Mi et al., 2017; Panno and Giordano, 2014; Wrześniok et al., 2017). These results suggest that if they can target cancer cells *in vivo*, furocoumarins could be a potential therapeutic agent for some cancers.

Complex photophysical, photochemical and biological mechanisms are based on photodynamic action through compounds, but it is obvious that the first stage is determined by the photophysical processes occurring in molecules and leading to the effective population of triplets. In practice, it is necessary to select the more suitable optimal compounds for a particular medical treatment, and to minimize their effects on the body. The effects of furocoumarin on human health remain complex, and there are still many questions regarding the safety of their medicinal use and their consumption with food.

## Objects and Methods of Research

### Objects

**FIGURE 1:**
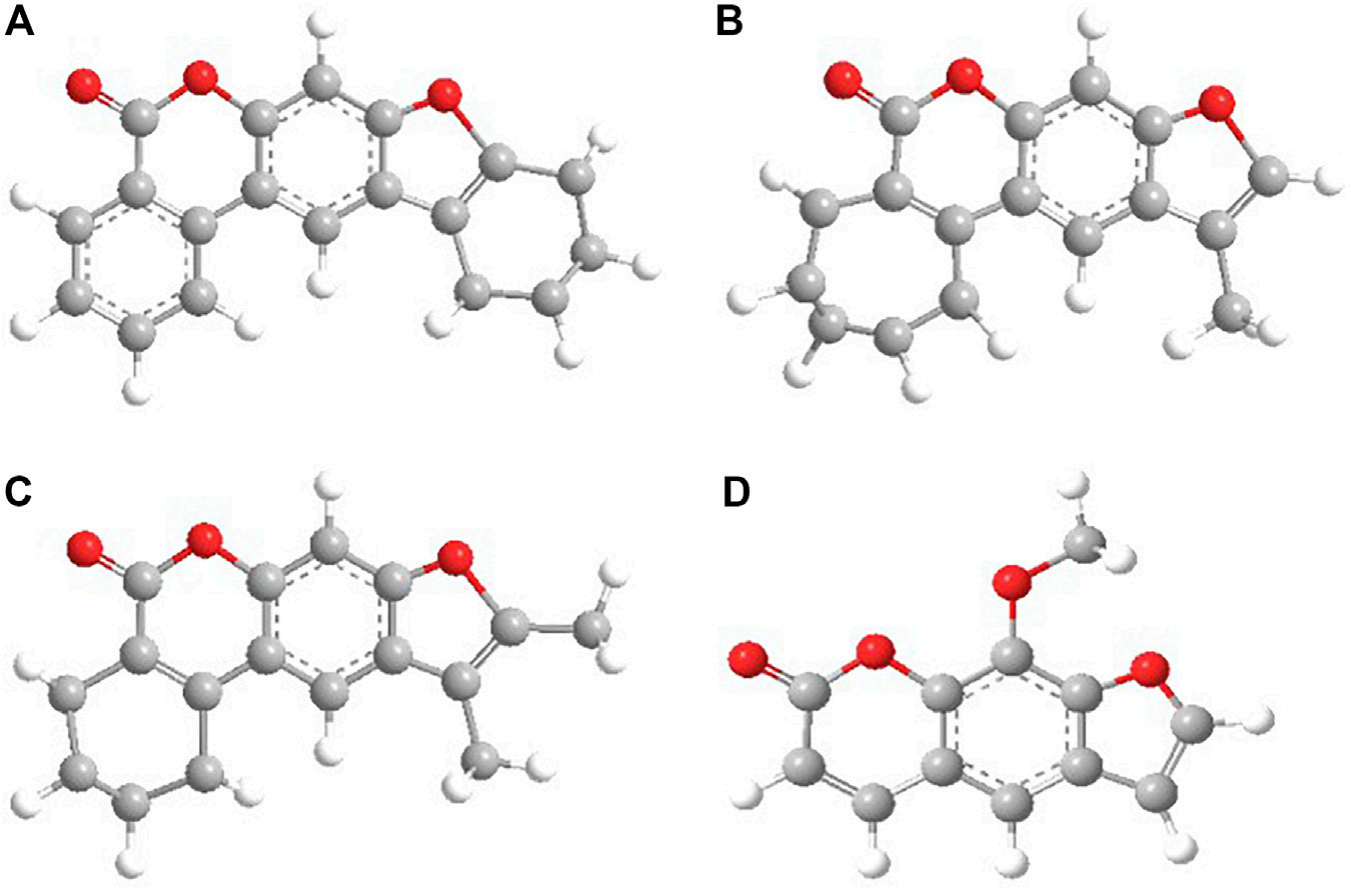
Chemical structures of the studied molecules: **(A)** KC3; **(B)** KC4; **(C)** KC5; **(D)** 8-MOP.

In the work, as objects of research, we used substances with chemical purity (99.8%) from Aldrich, Code: 56448: 8-methoxypsoralen (8-MOP); and also considered a number of objects for research with a chemical purity of 99.7% (Garazd et al., 2002; Garazd et al., 2006): 3,4-phenyl-4 ′, 5′-cyclohexylpsoralen (KC3), 4′-methyl, 3,4-cycloheptylpsoralen (KC4), 4′, 5′-dimethyl-3,4 -cyclohexylpsoralen (KC5). The structural formulas of all studied compounds are shown in Figure 1. We have given simple usual structures of the objects under study in the Supplementary Material.

Aldrich ethanol C_2_H_5_OH with chemical grade (99.9%) was used as a solvent. The experiments on absorption from the ground state, fluorescence, and induced absorption were carried out at room temperature 20°C and a pressure of 760 mm Hg. The phosphorescence experiment was carried out at a temperature of 77 K.

### Equipment

The obtained characteristics of 8-MOP were interpreted in comparison with the data for KC3, KC4, and KC5 molecules. The compounds under study are readily soluble in organic solvents and poorly soluble in water. The spectral-luminescent characteristics of the solutions were recorded on a CM2203 spectrofluorimeter (SOLAR, Belarus), an Evolution-600 spectrophotometer (Thermo Scientific), and a Cary Eclipse spectrofluorimeter (Varian) with an Optistat DN cryostat. To measure the spectra of induced absorption, we used a setup based on the pump-probe method.

### Research Methods

The study of the spectral-luminescent properties of substituted coumarins was carried out using an integrated approach combining experimental and theoretical consideration of the spectral-luminescent properties. The phosphorescence quantum yields were determined using the method of comparison with the standard. 8-MOP was chosen as a standard, emitting in a close spectral region; its phosphorescence quantum yield at 77 K in ethanol is 0.17 (Mantulin and Song, 1973). The concentration of the substances under study in solutions was selected such that the optical density at the excitation wavelength (330 nm) was 0.1.

To study the spectra of induced absorption, we used an experimental setup designed to record the spectra of nonstationary differential absorption by the pump-probe method with a fluorescent probe. The setup provided nanosecond time resolution. Its optical design, principle of operation and signal processing are given in (Svetlichnyi, 2010). The setup makes it possible to separate the absorption spectra of short-lived states and products, for example, singlet-singlet (S_1_ → S_n_) absorption and long-lived (longer than the pump pulse duration), for example, triplet-triplet (T_1_ → T_m_) absorption of molecules. Pumping was performed by the third (355 nm) harmonic of a pulsed Nd: YAG laser with Q-switching, τ_1/2_ = 7 ns, E_imp_ = 20÷40 mJ. The fluorescence of a mixture of dye solutions (range 350÷750 nm, luminescence duration 9 ns), excited by the same pump laser, was used as the probe radiation. The delay line was 10 m, which corresponds to a delay time of about 30 ns. The concentration of the investigated solutions for the experiment with a pumping probe was chosen to be 0.01 mM, while the optical density (OD) at the pump wavelength is in the range of 0.07÷0.18 relative units.

### The Photostability of the Compounds

To study of compounds photostability could provide information about their preservation. The solutions of substituted coumarins were irradiated in quartz cells with an optical layer thickness of 1 cm. As a source of UV radiation for photochemical studies, we used a U-type exciplex barrier discharge exciplex lamp based on working Xe and Cl* molecules (λ_em_ = 308 nm) with parameters Δλ = 5–10 nm, illumination power density 18 mW/cm^2^, volumetric energy dose 8.1 J/cm^3^, pulse repetition rate 200 kHz, pulse duration 1 μs. The intensity of the light source I = 4×10^15^ photons/s was determined by the method (Calvert and Pitts, 1966; Becker, 1976). The irradiation time varied from 2 to 32 min. Changes in the characteristics of substituted coumarins were monitored simultaneously by spectrophotometric and fluorescence methods on a CM2203 spectrofluorimeter (the device allows recording both fluorescence and absorption spectra). For experimental studies, ethanol solutions of the studied compounds with a concentration of 0.1 mM were prepared.

### Calculations

In the 60–70 s of the 20th century, a large number of semiempirical methods were created based on the zero differential overlap approximation. In semiempirical methods, the main part of the molecular integrals of the Coulomb repulsion is neglected. In addition, the core integrals are usually not calculated exactly, but are replaced by parameters that are calibrated so as to either obtain the best agreement between the calculated and experimental characteristics, or to achieve agreement with ab initio calculations, when the values of the group of physical properties and quantities calculated by this method are good enough. Most often, semiempirical methods use the valence approximation, according to which only electrons and the corresponding valence shell orbitals are taken into account in the LCAO MO expansion; internal electrons, for example, 1s carbon and other elements of the second and higher periods, are considered to be localized in the corresponding atomic orbitals and form an unpolarized core. Semiempirical methods are quite simple and are applied to the calculation of large molecules on modern computers.

In this work, a set of quantum-chemical programs is used, which makes it possible to correctly and reliably solve the assigned tasks and obtain a fairly good agreement (∼5÷10%) with the available experimental spectral data. And, most importantly, the software package that we have chosen made it possible to interpret the available experimental data and opened up the possibility of predicting the behavior of molecular structures in advance. Since theoretical studies of the molecular photonics of furocoumarins showed the low efficiency of the “standard” calculation methods, therefore, a set of quantum-chemical programs was used to study them, the basis of which is the semiempirical method of intermediate neglect of differential overlap (INDO) with original spectroscopic parametrization (Alfimov and Galeeva, 1986). The method has been successfully developed for a long time for the correct calculation of the spectral and luminescent properties of polyatomic organic molecules. Over the past 30 years, this package has been intensively used to study the photonics of polyatomic organic molecules. Using the software package, you can determine the important characteristics of the electronic states of polyatomic molecules: the energies and nature of molecular orbitals, the energies and wave functions of singlet and triplet electronically excited states, the oscillator strength and polarization of electronic transitions, the distribution of the electron density on the atoms and bonds of the molecule, the dipole moments in ground and excited states, rate constants of radiative and nonradiative processes involving electronically excited states of molecules, as well as absorption spectra from excited singlet and triplet states (Artyukhov et al., 2008; Alfimov et al., 2014; Artyukhov et al., 1997; Plotnikov, 1979; Pomogaev and Artyukhov, 2001). Moreover, this software set has achieved success in solving the problems of large molecules photonics (Plotnikov, 1979; Artyukhov and Pomogaev, 2000; Bocharnikova et al., 2019; Liano et al., 2003; Reveguk et al., 2020; Tchaikovskaya et al., 2020). The methodology for studying the spectral-luminescent properties of complex molecules was described in (Alfimov and Plotnikov, 2014; Bocharnikova et al., 2020). In order to construct a diagram of electronically excited states of furocoumarins, the geometry was optimized by the quantum-chemical method AustinModel 1 (Austin model 1 or AM1) (Dewar Michael, 2012; Quirante, 1995). The exact structural parameters of the studied molecules (bond lengths, bond and torsion angles) are unknown; therefore, the geometry of the ground state was carefully optimized by the method of molecular mechanics (MM2) from the popular Chem Office software program (Blatov et al., 2005). The ChemDraw Ultra was used to create a spatial model of the molecular structure. The geometry optimization method AM1 was determined using the Chem3D Ultra and HyperChem programs (ChemOffice, 2000; HyperChem 7.0, 2021).

The calculation of the Cartesian coordinates of atoms in furocoumarin molecules was performed using the moco02.exe program. As the initial data, quantitatively determining the spatial structure of the molecule, we used the values of the lengths of chemical bonds, bond and torsion angles (angles of rotation) (Artyukhov and Pomogaev, 2000). The indo02.exe program was used to calculate the electronic structure and spectra of polyatomic molecules by the INDO method. This made it possible to take into account only the valence electrons of the atoms that make up the molecule. The revue02.exe program was used to calculate the rate constants of intramolecular radiative and nonradiative processes and the quantum yield of fluorescence (Artyukhov and Pomogaev, 2000). The K_ST_.exe program was used to calculate the rate constants of the intercrossing conversion (Artyukhov and Pomogaev, 2000). Absorption spectra from excited states provide information on high-lying energy levels and photoprocesses involving such states (Artyukhov and Mayer, 2012; Artyukhov and Maier, 2001; Artyukhov and Galeeva, 1986).

## Results

### Spectral-Luminescent Characteristics

**FIGURE 2:**
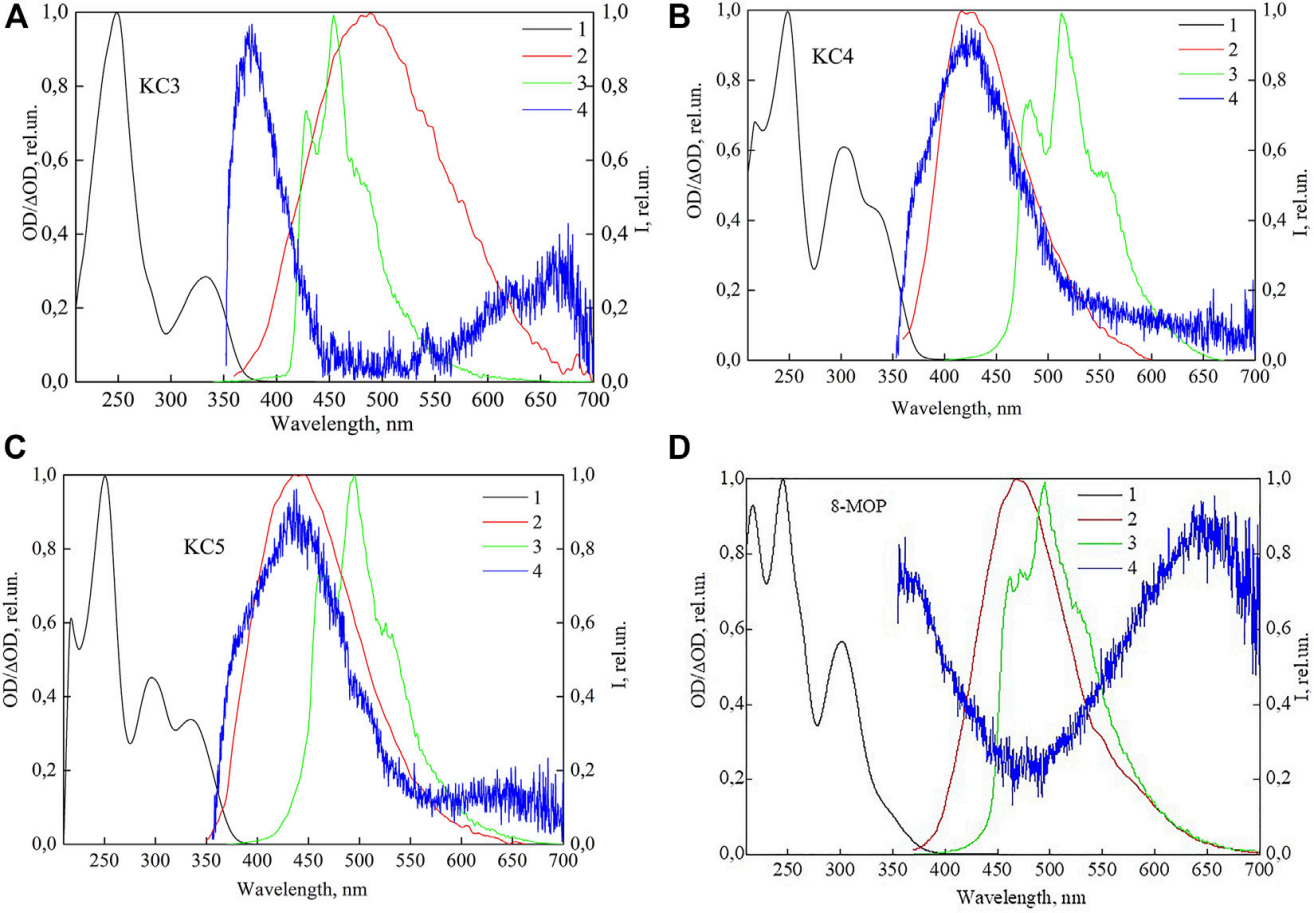
Normalized spectra: 1—absorption, 2—fluorescence, 3—phosphorescence, 4—induced absorption for KC3 **(A)**; KC4 **(B)**; KC5 **(C)**; 8-MOP **(D)**.

For the investigated series of molecules: KC3, KC4, KC5, 8-MOS (see Figure 1) spectral-luminescent characteristics were obtained. Figure 1 shows that 8-MOP has been a high electron localized in the S_0_ state than KC3 and KC5. In the Supplementary Material, changes in electron density redistribution for furocoumarins have been reported. The oxygen atom of the carbonyl group of 8-MOP, KC3, and KC4 plays an important role in charge transfer during the transition from the S_0_ to the S_3_ state upon excitation. The carbonyl group of furocoumarins participate in intermolecular interactions. Quantum-chemical calculations have shown that the oxygen atom of KC5 carbonyl group is less involved in the redistribution of the effective charge than 8-MOP. Figure 2 shows the fundamental laws, namely the normalized spectra: absorption, fluorescence, phosphorescence, induced absorption of the compounds under study.

Figure 2 shows that the maximum of the long-wavelength absorption band for compounds KC3 and KC5 is located at 332–334 nm (see Figures 2A,C), the maximum of the short-wavelength band for the entire group of compounds lies in the region of 245–250 nm, for 8-MOP the maximum of the long-wavelength band is at 302 nm (see Figure 2D). The maximum of the fluorescence spectra for the whole series of studied compounds falls on the 420–488 nm region: for compounds KC3 and KC5 is located at 488 and 438 nm (see Figures 2A,C); for KC4 and 8-MOP–at 422 and 468 nm (see Figure 2), respectively. The experimental maximum of phosphorescence for a substituted coumarins falls on the long-wavelength range of 455–513 nm. The maximum of the phosphorescence spectra for КС3 falls on 452 nm, for KC4—513 nm, for KC5—496 nm and for 8-MOP—495 nm, respectively (see Figure 2). The maximum of the spectra of induced absorption lies in the region of 370–435 nm. The maximum of the spectra of induced absorption lies for КС3 falls on 377 nm, for KC4—425 nm, for KC5—430 nm and for 8-MOP—360 nm, respectively (see Figure 2). The experimental data are consistent with the data obtained using quantum-chemical calculations (see Table 3). According to the data of quantum-chemical calculations, the appearance of these bands in the spectra is due to triplet-triplet transitions. However, in the spectra of induced absorption there are bands in the region of 650 nm. This band is very well registered for 8-MOS (see Figure 2D) and not explicitly for other molecules. It can be assumed that this streak is associated with the appearance of the spice.

### Diagrams of Electronically Excited States

**FIGURE 3:**
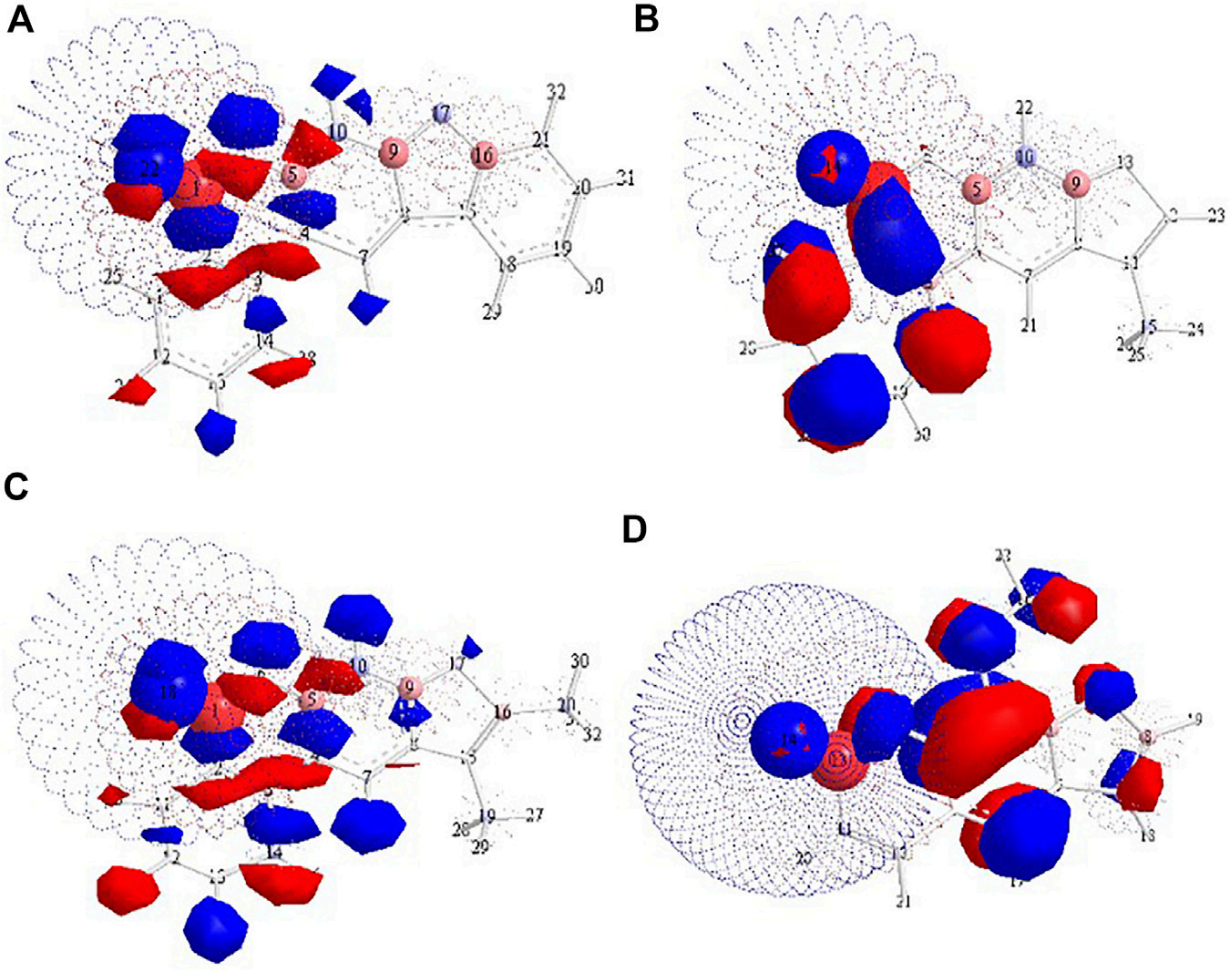
The structure of furocoumarins: **A**—KC3; **B**—KC4; **C**—KC5; **D**—8-MOP. The color corresponds to a partial charge. Atoms carrying a partial positive charge are colored red, and the negative charge is blue. Dimensions reflect the amount of charge. The dotted lines in the figures show the surfaces, the radius of which corresponds to the magnitude of the charge. The data are given for state S_0_.

Figure 3 demonstrates the distribution of the effective charge in molecules over fragments using quantum calculations. Delocalization of the effective charge was observed for the 8-MOS molecule. It is shown (see Table 1) that all the compounds studied absorb in one spectral region: the experimental long-wavelength band is located in the region of 332–334 nm. The nature of the first excited singlet state is of the ππ*-type for all compounds, and the second excited singlet is predominantly of the nπ*-type, or of a mixed type. There is some change in the dipole moment of the systems under study, depending on the structure of the studied substituted coumarin. The S_0_ → S_1_ transition occurs to states of the ππ * type, for which we observe the largest values of the dipole moments. The dipole moment of the singlet S_2_ states of the nπ*-type nature is less in comparison with the states of the ππ*-type nature. Comparing the experimental and theoretical values of the wavelengths for a number of studied compounds, we note good agreement between the data.

**TABLE 1:** Singlet-Singlet absorption spectra of several compounds.

Object	Quantum chemical calculations data					Experimental
	Transition	Energy, *E*_*i*_, cm^−1^ (nm)	Oscillator strength, *f*	Dipole moment, d	Orbital type of the transition	λ, nm
KC3	S_0_→S_1_	30700 (326)	0.46	8.29	ππ*	332
	S_0_→S_2_	33000 (303)	0.00	3.39	nπ*	-
	S_0_→S_5_	35100 (285)	0.13	8.47	ππ*	282
	S_0_→S_8_	39700 (252)	0.25	8.87	ππ*	246
KC4	S_0_→S_1_	30200 (331)	0.51	7.89	ππ*	332
	S_0_→S_2_	33300 (300)	0.12	7.45	nπ*+ππ*	301
	S_0_→S_7_	40300 (248)	0.47	8.62	ππ*	252
KC5	S_0_→S_1_	30200 (331)	0.39	9.54	ππ*	334
	S_0_→S_2_	33100 (302)	0.00	3.29	nπ*	-
	S_0_→S_3_	34100 (293)	0.09	8.53	ππ*+σπ*	296
	S_0_→S_7_	39100 (256)	0.33	9.04	πσ*	252
8-MOP	S_0_→S_1_	31700 (316)	0.08	8.92	ππ*	332
	S_0_→S_2_	33600 (297)	0.00	3.36	nπ*	301
	S_0_→S_3_	34100 (294)	0.19	6.56	ππ*	-
	S_0_→S_7_	40500 (245)	1.13	10.39	πσ*	245

Table 2 shows the data on luminescence and the values of Stokes shifts for all objects of this study.

**TABLE 2:** Luminescence quantum yields of the compounds.

Object	Fluorescence quantum yield of, φ_fl_		Experimental data			
	Theoretical	Experimental	Fluorescence wavelength, λ_fl_, nm	Stokes shift, cm^−1^	Phosphorescence	
					λ_ph_, nm	Quantum yield, φ_ph_
KC3	0,009	0,036	488	8900	452	0,71
KC4	0,031	0,029	422	6500	513	0,26
KC5	0,003	0,059	438	7600	496	0,45
8-MOP	0.001	0,0013	468, 470 Lai et al. (1982)	12200	495	0,19

### Stokes Shift

It can be seen from Table 2 that the values of fluorescence quantum yields obtained experimentally for a number of studied systems are in good agreement with the data of quantum-chemical calculations by the AM1 method. For all investigated substituted coumarins: KC3, KC4, KC5, 8-MOP, a significant Stokes shift of 6,500÷12200 cm^−1^ is observed. The largest value of the Stokes shift corresponds to the substance 8-MOP. This value can be associated with changes in the geometry of molecules in excited singlet states. From the experimental and theoretical data, it can be seen that all the investigated substituted coumarins are weakly fluorescent. It can be seen from the literature that for the known furocoumarins, the experimental values of the fluorescence quantum yields are in good agreement with the data for compounds with a related structure (for psoralen–φ_fl_ = 0.01 ÷ 0.023, for 8-MOP–φ_fl_ = 0.0013 (Mantulin and Song, 1973)). Compound KC3 has the highest phosphorescence quantum yield - 0.71. The information on the position of the bands and the quantum yields of fluorescence and phosphorescence of 8-MOP that we obtained is consistent with the literature data (λ_fl_ = 470 nm, λ_ph_ = 457 nm, φ_ph_ = 0.17) (Mantulin and Song, 1973; Lai et al., 1982).

Next, we studied the absorption spectra from excited states, due to the fact that they are informative from the point of view of obtaining data on the structure of energy levels and photoprocesses involving such states.

### Induced Absorption Spectra

Experimental induced absorption was found for a group of investigated compounds: KC3, KC4, KC5, 8-MOP, and theoretical T_1_-T_i_ absorption spectra were calculated by the quantum-chemical method (see Table 3). Analysis of the data shows that for all substituted furocoumarin molecules containing phenyl and cyclohexyl substituents (KC3), cycloheptyl and methyl radical (KC4), cyclohexyl and two methyl radicals (KC5), or methoxy group (8-MOP), T-T absorption spectra have some differences.

**TABLE 3:** Triplet-Triplet absorption spectra of several compounds.

Method	Compound							
	KC3		KC4		KC5		8-MOP	
	λ, nm (Т_1_-Т_i_)	Oscillator strength, *f*	λ, nm (Т_1_-Т_i_)	Oscillator strength, *f*	λ, nm (Т_1_-Т_i_)	Oscillator strength, *f*	λ, nm (Т_1_-Т_i_)	Oscillator strength, *f*
Calculation	377 (Т_1_-Т_24_)	0,08	425 (Т_1_-Т_13_)	0,014	430 (Т_1_-Т_15_)	0,01	471 (Т_1_-Т_12_)	0,01
	330 (Т_1_-Т_30_)	0,08	384 (Т_1_-Т_14_)	0,032	386 (Т_1_-Т_18_)	0,01	327 (Т_1_-Т_20_)	0,02
	317 (Т_1_-Т_32_)	0,21	321 (Т_1_-Т_20_)	0,225	331 (Т_1_-Т_23_)	0,19	300 (Т_1_-Т_24_)	0,26
Experiment	377	-	425	-	430	-	360	-

It can be seen from the table that the Т_1_-Т_13_, Т_1_-Т_15_, Т_1_-Т_12_ transitions for the KC4, KC5 and 8-MOP molecules, respectively, are recorded in a longer wavelength region of the spectrum compared to the T_1_-T_24_ KC_3_ transition.

It is known from the literature that for an unsubstituted furocoumarin molecule the absorption maximum in benzene coincides with the absorption maximum in water. According to the data of (Parrish et al., 1974; Bethea et al., 1999), when passing from methanol to benzene, the T-T absorption spectrum of furocoumarin shifts by 10 nm to the red region.

Experimentally, the spectra of induced absorption were obtained for all studied compounds, the maxima of which are at 374, 425, 438, and 456 nm for KC3, KC4, KC5, 8-MOS, respectively. Due to the fact that the probe radiation lies in the long-wavelength region of the spectrum (350–700 nm), the transitions T_1_-T_32_ for KC3, T_1_-T_20_ for KC4, T_1_-T_23_ for KC5 and T_1_-T_24_ for 8-MOP were not recorded with using an experimental setup.

Transitions T_1_-T_24_ and T_1_-T_30_ for connecting KC3, T_1_-T_13_ and T_1_-T_14_ for connecting KC4, T_1_-T_15_ and T_1_-T_18_ for connecting KC5, T_1_-T_12_ and T_1_-T_20_ for an 8-MOP connection are carried out between the states of one nature (ππ*-ππ*), and the transitions Т_1_-Т_32_, Т_1_-Т_20_, Т_1_-Т_23_, Т_1_-Т_24_ for compounds КС3, КС4, КС5, 8-MOP, respectively, between states of different orbital nature (ππ * - πσ *). This explains the reason for the more intense triplet-triplet absorption in the region of 317–331 nm. Comparing the results of experimental and quantum-chemical studies, we note good agreement for the long-wavelength T_1_-T_24_, T_1_-T_13_, T_1_-T_15_, and T_1_-T_12_ transitions of substituted coumarins KC3, KC4, KC5, and 8-MOP, respectively.

### The Channels of Degradation of the Excitation Energy

Due to the fact that compounds fluoresce weakly, it is considered what happens with the energy further, for this, the rate constants of radiation, internal and intersystem conversions were calculated using the quantum-chemical method. The values of the rate constants for the photophysical processes occurring in the compounds under study are shown in Table 4.

**TABLE 4:** Rate constants of photophysical processes^a^ of lower singlet excited states. The geometry of the molecular structure was optimized by the AM1.

Compound	k_r_, s^−1^	k_IC_, s^−1^	k_ST_, s^−1^
8-MOP	5.7×10^7^	5.8×10^3^	3.4×10^10^
KC3	1.4×10^8^	1.4×10^3^	1×10^10^
KC4	1.4×10^8^	2.7×10^3^	1×10^9^
KC5	1.0×10^8^	3.4×10^3^	1×10^9^

a Where ${\text{k}}_{\text{r}}$ , is the rate constant of radiation; ${\text{k}}_{\text{IC}}$ , is the rate constant of internal conversion; ${\text{k}}_{\text{ST}}$ , is the rate constant of the intersystem crossing process.

It was found that, for the entire series of substituted coumarin studied, there is an effective intercombination conversion with large rate constants ∼ 10^10^÷10^11^ s^−1^ due to the close arrangement of levels having different orbital nature of the ππ* and πσ* types, which led to small values of quantum yields fluorescence and, consequently, to a significant population of the triplet state.

**FIGURE 4:**
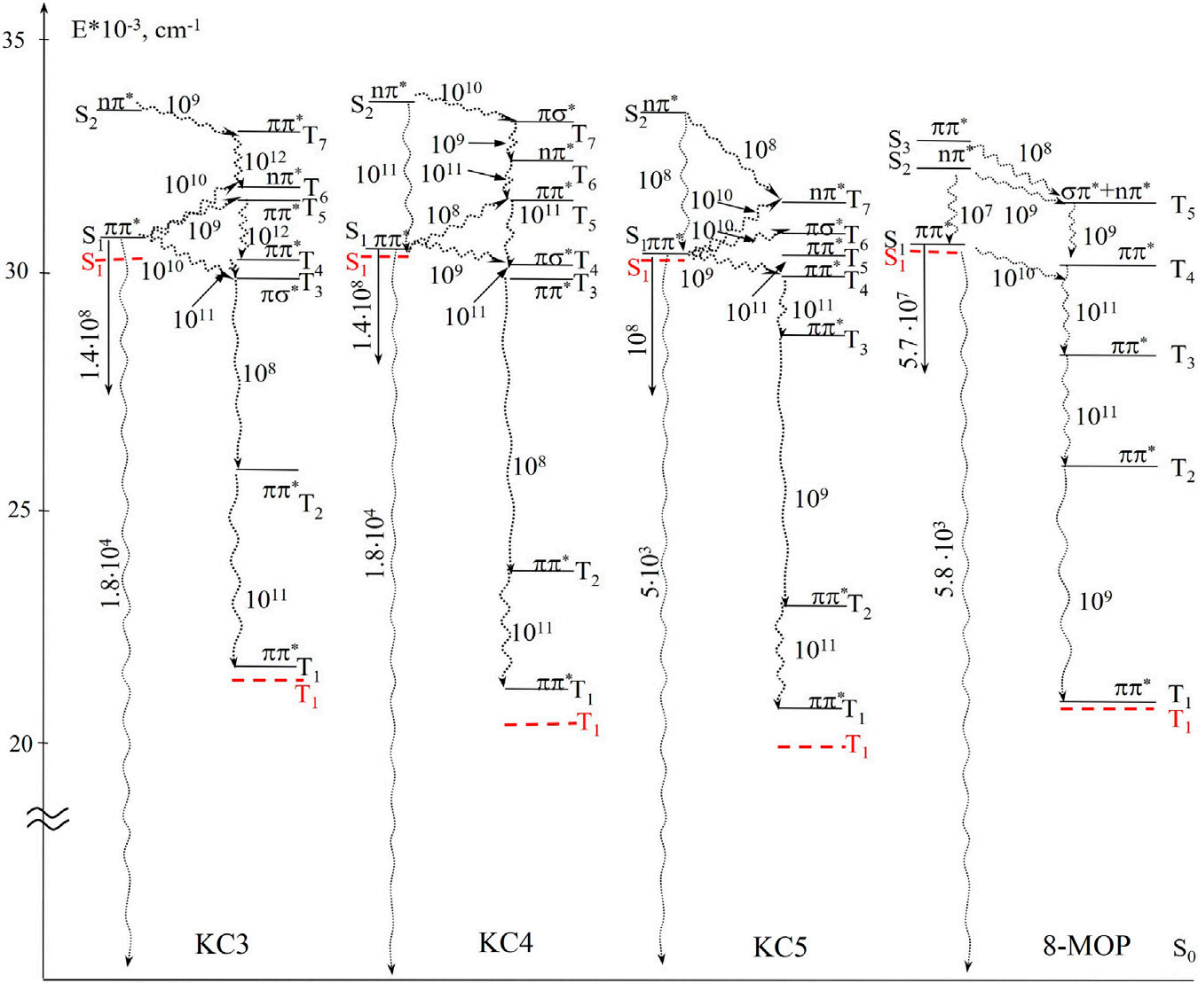
Diagrams of electronically excited states of the compounds (dashed lines indicate experimental values). Arrows show the ways of energy relaxation, the numbers near them are the rate constants of these processes, s^−1^.

After optimization of the geometry by the AM1 method (see Figure 4) and quantum-chemical calculation, schemes of electronically excited states were constructed and the rate constants of photophysical processes were estimated. From the results obtained, we can say that there is good agreement between the calculated and experimental data. Let’s consider the ways of deactivation of energy in more detail.

Energy level diagrams represent the main processes that occur in molecules after absorption of radiation. The figure shows the most effective rate constants of internal and intercombination conversions, as well as the radiation constants. An analysis of the electronically excited state diagrams showed that, for the compounds studied, the lower singlet electronically excited state S_1_ has the orbital nature of the ππ*-type and the S_2_ state, predominantly of the nπ*-type. For the group of compounds under study, the singlet S1 is located in the region of 316–331 nm, which is in good agreement with the experimental data (they are shown by dashed lines): 331–334 nm (see Table 1) Electronic singlet-singlet transitions of the ππ*-type in the lower singlet excited state S_1_ and in the S_3_ state are formed mainly by carbon atoms, and the nπ*-type transitions are formed by carbonyl oxygen atoms for the entire group of the studied substituted coumarins. The low value of the quantum yield of fluorescence is determined by the high values of the rate constants of nonradiative processes in comparison with radiative decay. This explains the high values of phosphorescence quantum yields (0.19–0.71), which were obtained as a result of experimental studies at the facility.

The intersystem conversion process is the main channel for degradation of the excitation energy (k_ST_ ∼ 10^10^ s^– 1^) for the entire group of substituted coumarins. The high value of the intersystem conversion constant is due to the close location of the ππ*-type singlet state and the πσ*-type triplet state. Figure 3 shows that the channels of degradation of the excitation energy in the 8-MOP complex have one main channel through the system of triplet states, following one after the other:

$${\text{S}}_{1}\text{* }\to {\text{ T}}_{4}\text{* }\to {\text{ T}}_{3}\text{* }\to {\text{ T}}_{2}\text{* }\to {\text{ T}}_{1}\text{*}\text{.}$$

KC3 has the following main channel for degradation of the excitation energy:

$${\text{S}}_{1}\text{* }\to {\text{ T}}_{3}\text{* }\to {\text{ T}}_{2}\text{* }\to {\text{ T}}_{1}\text{*}\text{.}$$

KC4 has one main channel for degradation of the excitation energy:

$${\text{S}}_{1}\text{* }\to {\text{ T}}_{4}\text{* }\to {\text{ T}}_{3}\text{* }\to {\text{ T}}_{2}\text{* }\to {\text{ T}}_{1}\text{*}\text{.}$$

KC5 has the following main channel for degradation of the excitation energy:

$${\text{S}}_{1}\text{* }\to {\text{ T}}_{6}\text{* }\to {\text{ T}}_{5}\text{* }\to {\text{ T}}_{4}\text{* }\to {\text{ T}}_{3}\text{* }\to {\text{ T}}_{2}\text{* }\to {\text{ T}}_{1}\text{*}\text{.}$$

It can be seen from the diagram that for the investigated series of substituted coumarins, the nature of the T_1_ state is of the ππ*-type. Taking into account the data (nature and rate of intersystem crossing conversion), it can be concluded that the studied compounds are effective ππ*-sensitizers. For the studied compounds, good agreement was obtained between the calculated and experimental data for all interpreted bands, which confirms the possibility of using the calculation scheme for interpreting the spectra.

### Photostability

Quantum yields of photodegradation calculated by the formula (Becker, 1976):

$$\gamma =\frac{\frac{D_{u}-D_{i}}{D_{u}}\cdot C\cdot 10^{-3}N_{A}}{I\cdot t}$$

where D_u_ and D_i_–optical density of unirradiated and irradiated solutions, rel. un.; С–concentration of solution, M; N_А_–Avogadro’s number, 6,022·10^23^ mole^−1^; I–irradiation intensity, photon/s; t–exposure time, s.

Due to the fact that photostability is an important property for a wide variety of applications, we studied the effect of radiation from a lamp source on the spectral-luminescent properties of a number of substituted coumarins (see Table 5). The dependence of the optical density on the irradiation time is obtained. Analysis of the data in Table 5 showed that, after 30 min of excilamp irradiation, the highest phototransformation efficiency was recorded for KC3. The optical absorption density for KC3 dropped from 0.750 to 0.343. This indicates that more than 50 percent of the KC3 molecules are photodegraded. However, it should be noted that the values of the quantum yield of photodecay turned out to be low–less than 0,01 and did not give correct agreement with the number of decayed molecules.

**TABLE 5:** Absorbance intensity and photodecay quantum yield of compounds under the irradiation of XeCl* excilamp.

Irradiation time, min	Compounds			
	KC3	KC4	KC5	8-MOP
	Absorbance intensity, relative units			
0	0.750	0.713	0.738	0.733
2	0.697	0.688	0.678	0.704
4	0.653	0.668	0.625	0.688
8	0.591	0.603	0.550	0.646
16	0.485	0.523	0.467	0.568
32	0.343	0.438	0.385	0.472
Photodecay quantum yield	0.004	0.007	0.006	0.004

The effect of XeCl* excilamp irradiation on the photostability of the investigated series of substituted furocoumarins was evaluated. Note that the change in optical density was recorded at the wavelength of the absorption maximum of the unirradiated solution, namely: the wavelength of the absorption maximum for KC3 and KC4 is 332 nm, for KC5 − 334 nm, and for 8-MOP − 302 nm. Radiation of 308 nm falls into the region of long-wave absorption of the entire group of compounds studied, more precisely, into the S_2_-state. Therefore, upon irradiation with an excilamp, direct photolysis of the molecule can occur in the system. The calculated quantum yields of photodegradation of a number of substituted furocoumarin show that all compounds are highly photostable. The mechanism of photosolvolysis is known from the literature, according to which the primary stage is a nucleophilic attack of a solvent molecule on 4 ′or 5′ carbon atoms of furocoumarin in an excited state (Caffieri, 2002). In the case of KC3, KC4, and 8-MOP, the methyl and methoxy groups sterically hinder the addition of the solvent and reduce the reactivity of the substrate due to an increase in the electron density at the reaction center. As for the KC3 compound, it is also possible for it to break the 4′-5 ′bond.

## Conclusion

Based on the data obtained as a result of optimization of the geometry of molecules by the AM1 method and quantum-chemical calculation by the NPDP/s method, a diagram of the electronically excited states of the compounds under study is constructed. Thanks to this scheme, it was found that a number of substituted coumarins are characterized by effective intersystem crossing (∼10^10^÷10^11^ s^−1^). The presence of such constants is explained by the close arrangement of levels with different orbital nature of the ππ* and nπ* -types. The values of the quantum yields of fluorescence (0.0013–0.059) and phosphorescence (∼0.19÷0.71) were determined, which were explained using the scheme of electronically excited states. The triplet-triplet absorption spectra of the studied series of substituted coumarin are calculated. The states involved in the formation of these spectra have been established. The calculated quantum yields of photodegradation of a number of substituted furocoumarin show that all compounds have high photostability (0.004÷0.007). It should be noted that the calculated data are in good agreement with the data obtained during the experiments. The main channel for deactivation of the excitation energy in a number of substituted coumarins is singlet-to-triplet state conversion and then a decay through a system of triplet states in 8-MOP: S_1_* → T_4_* → T_3_* → T_2_* → T_1_*; in КС3: S_1_* → T_3_* → T_2_* → T_1_*; in КС4: S_1_* → T_4_* → T_3_* → T_2_* → T_1_*; in KC5: S_1_* → T_6_* → T_5_* → T_4_* → T_3_* → T_2_* → T_1_*.

## Data Availability

The original contributions presented in the study are included in the article/Supplementary Material, further inquiries can be directed to the corresponding author.
