# Supplementary material for: Migration of Excitation Energy in Furocoumarins
Source: Front Chem. 2021 Nov 4;9:754950. doi: 10.3389/fchem.2021.754950 (PMC8600316; doi:10.3389/fchem.2021.754950)
Supplement: Supplementary file 1 [file DataSheet1.PDF]

Supplementary Material

1 Supplementary Tables

Structural formulas of furocoumarins, the effective charges (q, e) in the singlet states using the AM1 method:

8-MOП

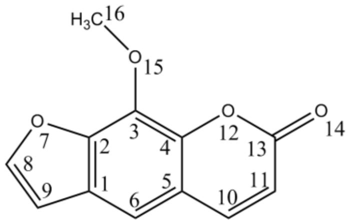

Atom	S_0	S_1	S_2	S_3
1	-0.038	-0.064	-0.105	-0.055
2	0.104	0.237	0.093	0.165
3	0.163	0.182	0.070	0.209
4	0.158	0.153	0.087	0.125
5	-0.044	-0.016	-0.086	-0.052
6	-0.055	-0.076	-0.187	-0.002
7	-0.225	-0.205	-0.232	-0.220
8	0.130	0.135	0.093	0.096
9	-0.064	-0.053	-0.090	-0.074
10	-0.036	-0.160	-0.141	-0.123
11	-0.048	-0.136	-0.012	-0.122
12	-0.167	-0.122	-0.107	-0.124
13	0.644	0.576	0.579	0.608
14	-0.611	-0.629	-0.075	-0.610
15	-0.287	-0.211	-0.294	-0.205
16	0.178	0.181	0.179	0.181
17	0.037	0.037	0.038	0.037
18	0.039	0.039	0.039	0.038
19	0.020	0.020	0.020	0.019
20	0.036	0.036	0.041	0.036
21	0.033	0.033	0.055	0.033
22	0.013	0.013	0.014	0.013
23	0.011	0.014	0.011	0.014
24	0.011	0.014	0.011	0.014

KC3

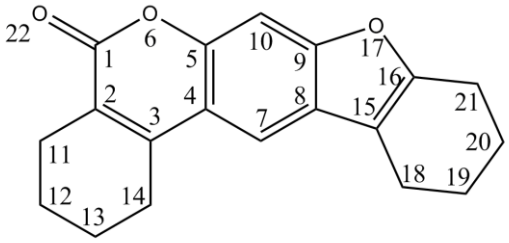

Atom	S_0	S_1	S_2	S_3
1	0.643	0.599	0.594	0.610
2	-0.037	-0.060	0.059	-0.031
3	0.011	-0.016	0.019	-0.007
4	-0.065	0.008	-0.080	-0.044
5	0.182	0.246	0.173	0.165
6	-0.182	-0.160	-0.098	-0.173
7	-0.044	-0.142	-0.111	-0.025
8	-0.010	0.014	-0.044	-0.014
9	0.0165	0.205	0.117	0.159
10	-0.072	-0.125	-0.110	-0.051
11	-0.007	-0.016	-0.056	-0.014
12	-0.020	-0.004	-0.070	-0.018
13	-0.013	-0.055	-0.107	-0.029
14	-0.045	-0.039	-0.094	-0.039
15	-0.030	-0.014	-0.055	-0.007
16	0.196	0.208	0.185	0.203
17	-0.231	-0.205	-0.233	-0.198
18	-0.034	-0.038	-0.050	-0.058
19	-0.021	-0.021	-0.036	-0.017
20	-0.036	-0.021	-0.074	-0.038
21	-0.038	-0.047	-0.054	-0.060
22	-0.607	-0.608	-0.195	-0.606
23	0.041	0.041	0.043	0.041
24	0.033	0.033	0.035	0.033
25	0.048	0.048	0.049	0.048
26	0.022	0.022	0.028	0.022
27	0.026	0.026	0.029	0.026
28	0.026	0.026	0.032	0.026
29	0.027	0.027	0.029	0.027
30	0.019	0.019	0.020	0.019
31	0.026	0.026	0.026	0.026
32	0.026	0.026	0.028	0.026

KC4

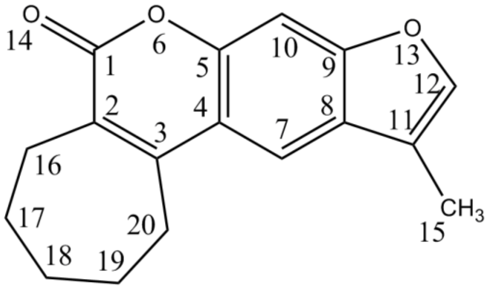

Atom	S_0	S_1	S_2	S_3
1	0.632	0.585	0.594	0.637
2	-0.080	-0.097	0.053	-0.078
3	0.006	-0.079	0.017	-0.126
4	-0.063	-0.052	-0.042	-0.149
5	0.178	0.192	0.137	0.269
6	-0.174	-0.162	-0.090	-0.102
7	-0.059	-0.059	-0.136	-0.039
8	-0.012	-0.008	-0.080	-0.069
9	0.151	0.137	0.163	0.195
10	-0.042	-0.049	-0.127	-0.004
11	-0.102	-0.037	-0.049	-0.019
12	0.184	0.185	-0.064	-0.008
13	-0.230	-0.191	-0.127	-0.029
14	-0.613	-0.607	-0.075	-0.086
15	-0.001	0.004	-0.074	-0.051
16	0.004	0.006	0.066	0.275
17	0.003	0.008	-0.241	-0.204
18	0.030	0.030	-0.194	-0.598
19	0.035	0.035	0.002	-0.027
20	0.029	0.029	0.041	0.028
21	0.017	0.020	0.034	0.026
22	0.013	0.013	0.028	0.022
23	0.017	0.020	0.050	0.044
24	0.006	0.006	0.035	0.021
25	0.018	0.016	0.027	0.020
26	0.018	0.016	0.019	0.013
27	0.000	0.000	0.013	0.020
28	0.016	0.018	0.005	0.001
29	0.016	0.019	0.013	0.019
30	0.004	0.009	0.002	0.006
31	0.004	0.008	0.000	0.005

KC5

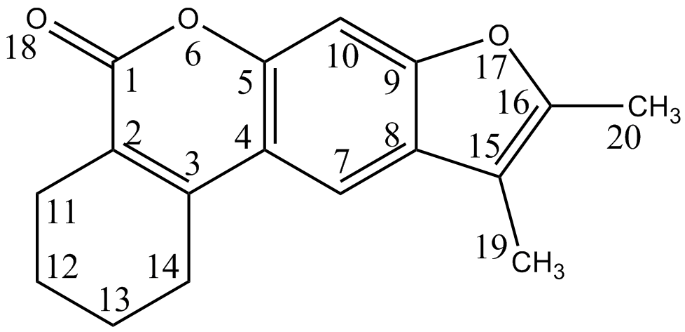

Atom	S_0	S_1	S_2	S_3
1	0.643	0.586	0.622	0.622
2	-0.041	-0.096	0.006	-0.078
3	0.010	-0.040	-0.018	-0.121
4	-0.049	-0.036	-0.065	-0.146
5	0.157	0.217	0.140	0.258
6	-0.164	-0.135	-0.101	-0.118
7	-0.057	-0.055	-0.145	-0.016
8	-0.031	-0.013	-0.069	-0.054
9	0.181	0.181	0.168	0.181
10	-0.069	-0.079	-0.102	-0.030
11	0.002	-0.056	-0.028	-0.019
12	-0.022	-0.030	-0.056	-0.027
13	-0.011	-0.092	-0.094	-0.027
14	-0.046	-0.086	-0.051	-0.089
15	-0.062	0.035	-0.085	-0.038
16	0.158	0.243	0.114	0.290
17	-0.228	-0.196	-0.230	-0.207
18	-0.618	-0.626	-0.242	-0.603
19	0.000	0.004	-0.004	-0.023
20	0.001	0.007	0.001	0.001
21	0.037	0.036	0.033	0.030
22	0.031	0.031	0.030	0.026
23	0.020	0.020	0.020	0.019
24	0.046	0.046	0.044	0.042
25	0.027	0.027	0.029	0.021
26	0.027	0.026	0.024	0.022
27	0.015	0.022	0.015	0.022
28	0.005	0.006	0.004	0.004
29	0.010	0.013	0.011	0.013
30	0.013	0.021	0.013	0.024
31	0.014	0.016	0.013	0.016
32	0.003	0.005	0.002	0.005

Atomic coordinates angles and bond lengths for the studied molecules:

1. 3,4-phenyl-4',5'-cyclohexylpsoralen (KC3).

Atom number	X	Y	Z
1	.000000	.000000	.000000
2	.000000	1.400000	.000000
3	-1.212436	2.100000	.000000
4	-2.424871	1.400000	.000000
5	-2.424871	.000000	.000000
6	-1.212436	-.700000	.000000
7	-3.722929	1.924449	.000000
8	-4.581999	.844450	.000000
9	-3.846736	-.287755	.000000
10	1.225426	-.707500	.000000
11	2.420541	-.017500	.000000
12	2.397608	1.442320	.000000
13	1.189095	2.087606	.000000
14	3.444448	2.068840	.000000
15	-1.212436	3.190000	.000000
16	-1.364134	-1.779392	.000000
17	-6.071942	.831447	.000000
18	-4.591566	-1.578023	.023170
19	-6.678771	-.478745	.474925
20	-6.074175	-1.659634	-.266941
21	1.250034	-2.117285	.000000
22	2.474501	-2.796019	.000000
23	3.674536	-2.074965	.000000
24	3.650102	-.675179	.000000
25	-6.435987	1.135134	-1.003666
26	-6.476220	1.573381	.719857
27	-7.755958	-.495993	.207583
28	-6.650917	-.530285	1.583378
29	-6.271819	-1.543670	-1.353030
30	-6.578707	-2.599474	.040058
31	-4.168792	-2.315084	-.691045
32	-4.558602	-2.038289	1.032709

Initial atom coordinates: X= .000000, Y= .000000, Z= .000000

Bonds		Bond length	Valence angle	Torsion angle
Start	Finish			
1	2	1.400000	90.000000	90.000000

2	3	1.400000	120.000000	90.000000
3	4	1.400000	120.000000	.000000
4	5	1.400000	120.000000	.000000
5	6	1.400000	120.000000	.000000
4	7	1.400000	128.000000	180.000000
7	8	1.380000	106.500000	180.000000
8	9	1.350000	108.500000	.000000
1	10	1.415000	90.000000	330.000000
10	11	1.380000	120.000000	90.000000
11	12	1.460000	119.100000	.000000
12	13	1.370000	119.000000	.000000
12	14	1.220000	120.000000	180.000000
3	15	1.090000	120.000000	180.000000
6	16	1.090000	112.000000	180.000000
8	17	1.490000	129.000000	180.000000
9	18	1.490000	117.000000	179.000000
17	19	1.520000	114.000000	200.000000
19	20	1.520000	111.000000	309.000000
10	21	1.410000	121.000000	270.000000
21	22	1.400000	120.000000	180.000000
22	23	1.400000	120.000000	360.000000
23	24	1.400000	120.000000	.000000
17	25	1.110000	109.000000	73.000000
17	26	1.110000	111.000000	316.000000
19	27	1.110000	109.000000	194.000000
19	28	1.110000	110.000000	79.000000
20	29	1.110000	109.000000	300.000000
20	30	1.110000	110.000000	184.000000
18	31	1.110000	112.000000	136.000000
18	32	1.110000	111.000000	256.000000

2. 4'-methyl, 3,4-cycloheptylpsoralen (KC4).

Atom number	X	Y	Z
1	.000000	.000000	.000000
2	.000000	1.400000	.000000
3	-1.212436	2.100000	.000000
4	-2.424871	1.400000	.000000
5	-2.424871	.000000	.000000
6	-1.212436	-.700000	.000000
7	-3.630210	1.886989	.000000
8	-4.479823	.799534	.000000
9	-3.739114	-.341058	.000000
10	1.225426	-.707500	.000000
11	2.403220	-.027500	.000000
12	2.380131	1.442319	.000000

13	1.171617	2.087605	.000000
14	3.426970	2.068839	.000000
15	-1.212436	3.190000	.000000
16	-1.401712	-1.773440	.000000
17	-4.336859	-1.683868	.021516
18	1.174690	-2.203792	.092387
19	2.294379	-2.835667	.903207
20	3.549099	-3.091715	.084355
21	3.938925	-1.938911	-.826401
22	3.746739	-.577996	-.177267
23	-5.567168	.875568	.000000
24	1.161850	-2.646776	-.925307
25	.262235	-2.668825	.520489
26	1.938178	-3.800540	1.320630
27	2.528158	-2.203216	1.784940
28	3.423896	-4.011223	-.524683
29	4.392083	-3.308751	.773103
30	3.329713	-1.941464	-1.754277
31	4.983380	-2.088096	-1.171306
32	4.438285	.137429	-.669234

Initial atom coordinates: X= .000000, Y= .000000, Z= .000000

Bonds		Bond length	Valence angle	Torsion angle
Start	Finish			
1	2	1.400000	90.000000	90.000000
2	3	1.400000	120.000000	90.000000
3	4	1.400000	120.000000	.000000
4	5	1.400000	120.000000	.000000
5	6	1.400000	120.000000	.000000
4	7	1.300000	128.000000	180.000000
7	8	1.380000	106.000000	180.000000
8	9	1.360000	109.000000	.000000
1	10	1.415000	90.000000	330.000000
10	11	1.360000	120.000000	90.000000
11	12	1.470000	119.100000	.000000
12	13	1.370000	119.000000	.000000
12	14	1.220000	120.000000	180.000000
3	15	1.090000	120.000000	180.000000
6	16	1.090000	110.000000	180.000000
9	17	1.470000	123.000000	179.000000
10	18	1.500000	118.000000	266.000000
18	19	1.520000	115.000000	216.000000
19	20	1.520000	113.000000	274.000000
20	21	1.520000	114.000000	44.000000
21	22	1.520000	113.000000	40.000000

8	23	1.090000	124.000000	180.000000
18	24	1.110000	110.000000	92.000000
18	25	1.110000	118.000000	338.000000
19	26	1.110000	109.000000	153.000000
19	27	1.110000	110.000000	38.000000
20	28	1.110000	110.000000	281.000000
20	29	1.110000	109.000000	166.000000
21	30	1.110000	111.000000	280.000000
21	31	1.110000	109.000000	165.000000
22	32	1.110000	108.000000	155.000000

3. 4′,5′-dimethyl-3,4-cyclohexylpsoralen (KC5).

Atom number	X	Y	Z
1	.000000	.000000	.000000
2	.000000	1.400000	.000000
3	-1.212436	2.100000	.000000
4	-2.424871	1.400000	.000000
5	-2.424871	.000000	.000000
6	-1.212436	-.700000	.000000
7	-3.722929	1.924449	.000000
8	-4.581999	.844450	.000000
9	-3.851271	-.302562	.000000
10	1.225426	-.707500	.000000
11	2.420541	-.017500	.000000
12	2.397451	1.452319	.000000
13	1.188937	2.097605	.000000
14	3.444290	2.078839	.000000
15	-1.212436	3.190000	.000000
16	-1.364134	-1.779392	.000000
17	-6.050600	.908570	.000000
18	-4.460712	-1.640105	.021516
19	1.199247	-2.207272	.000000
20	2.493106	-2.848190	.474925
21	3.689416	-2.274713	-.266941
22	3.723334	-.800183	.023202
23	.884659	-2.544841	-1.009545
24	.442422	-2.573761	.724570
25	2.482150	-3.925459	.207583
26	2.545357	-2.821694	1.583378
27	3.568319	-2.469253	-1.353030
28	4.615727	-2.803674	.040058
29	4.454073	-.341840	-.675398
30	4.173275	-.723067	1.034985
31	-6.414103	.924441	-1.027479
32	-6.452916	.035465	.513740

Initial atom coordinates: X= .000000, Y= .000000, Z= .000000

Bonds		Bond length	Valence angle	Torsion angle
Start	Finish			
1	2	1.400000	90.000000	90.000000
2	3	1.400000	120.000000	90.000000
3	4	1.400000	120.000000	.000000
4	5	1.400000	120.000000	.000000
5	6	1.400000	120.000000	.000000
4	7	1.400000	128.000000	180.000000
7	8	1.380000	106.500000	180.000000
8	9	1.360000	109.000000	.000000
1	10	1.415000	90.000000	330.000000
10	11	1.380000	120.000000	90.000000
11	12	1.470000	119.100000	.000000
12	13	1.370000	119.000000	.000000
12	14	1.220000	120.000000	180.000000
3	15	1.090000	120.000000	180.000000
6	16	1.090000	112.000000	180.000000
8	17	1.470000	126.000000	180.000000
9	18	1.470000	123.000000	179.000000
10	19	1.500000	119.000000	270.000000
11	22	1.520000	119.000000	179.000000
19	20	1.520000	114.000000	200.000000
20	21	1.520000	111.000000	309.000000
19	23	1.110000	108.000000	73.000000
19	24	1.110000	110.000000	316.000000
20	25	1.110000	109.000000	194.000000
20	26	1.110000	110.000000	79.000000
21	27	1.110000	109.000000	300.000000
21	28	1.110000	110.000000	184.000000
22	29	1.110000	110.000000	138.000000
22	30	1.110000	109.000000	254.000000
17	31	1.090000	109.500000	90.000000
17	32	1.090000	109.500000	210.000000

4. 8-methoxypsoralen (8-MOP).

Atom number	X	Y	Z
1	.000000	.000000	.000000
2	-.011036	1.452040	-.021293
3	1.225436	2.172133	.000222
4	2.416291	1.360490	.038970
5	2.378535	.060263	.049394
6	1.111369	-.726543	.026502

7	3.699383	1.857086	.100417
8	4.518987	.689018	.128241
9	3.790115	-.426466	.098538
10	-1.365479	-.640620	-.024085
11	-2.471175	.125963	-.066431
12	-1.139617	2.252085	-.059597
13	-2.328697	1.634525	-.088585
14	-3.323963	2.317907	-.130022
15	1.143383	3.554085	-.005600
16	2.392384	4.246085	.028396
17	1.098321	-1.826486	.033546
18	4.146418	-1.467119	.109242
19	5.610210	.821904	.168301
20	-3.467107	-.340713	-.083793
21	-1.457962	-1.736560	-.006631
22	2.087383	5.323086	.004396
23	2.946386	4.005087	.967395
24	3.008382	3.982083	-.864605

Initial atom coordinates: X= .000000, Y= .000000, Z= .000000

Bonds		Bond length	Valence angle	Torsion angle
Start	Finish			
1	2	1.452238	89.159885	90.435459
2	3	1.431035	119.758304	268.527100
3	4	1.441666	115.520240	.165080
4	5	1.300817	122.613834	.220974
5	6	1.491743	123.486045	359.823499
4	7	1.377210	124.595034	181.825077
7	8	1.427203	103.932972	179.590429
8	9	1.332832	111.745637	359.462999
1	10	1.508478	89.085154	205.133812
10	11	1.346108	120.145058	271.553439
2	12	1.383920	125.776491	88.650427
11	13	1.515437	119.349212	359.958586
13	14	1.208007	119.090159	179.813733
3	15	1.384398	116.810025	180.755147
15	16	1.428293	115.576044	179.459574
6	17	1.100043	122.504757	179.863073
9	18	1.100011	127.909546	179.989083
8	19	1.100014	118.133252	179.411571
11	20	1.099986	120.171641	180.012935
10	21	1.099974	119.932280	91.576207
16	22	1.119612	103.139635	179.339895
16	23	1.116565	110.446136	298.185773
16	24	1.116513	110.417769	60.522481
